# AI ethics in hospitality and tourism: theoretical perspectives, ethical beliefs, and actionable outcomes

**DOI:** 10.3389/frai.2026.1913343

**Published:** 2026-08-14

**Authors:** Nasim Binesh, Ahmad Syah

**Affiliations:** Department of Tourism, Hospitality & Event Management, University of Florida, Gainesville, FL, United States

**Keywords:** AI, epistemology, ethical beliefs, ethics, hospitality, tourism

## Abstract

**Introduction:**

As AI becomes embedded across hospitality and tourism, its ethical implications remain fragmented and largely undertheorized; this scoping review synthesizes the evidence on AI ethics in the sector.

**Methods:**

Following a PRISMA-guided search and screening of Web of Science, Scopus, and Emerald, we reviewed 32 studies and organized findings into nine cross-cutting themes, with associated research gaps and future questions. To extend prior descriptive work, we interpret the literature through epistemology and the ethics of belief, examining how AI systems in service contexts form, justify, and act upon “beliefs” (e.g., inferred preferences, risk scores, and recommendations) and how unjustified beliefs can generate ethical harms.

**Results:**

We map ethical concerns across key hospitality and tourism sub-sectors (e.g., accommodation, transportation, entertainment) and translate them into actionable guidance. Specifically, we propose (1) a sectoral risk framework that classifies common AI applications from unacceptable to minimal risk, and (2) a structured AI life-cycle approach identifying ethical safeguards at problem definition, data collection, model development, deployment, and feedback.

**Conclusion:**

The review advances a theory-based understanding of AI ethics in human-centric service industries, offering practical implications for managers, policymakers, and researchers seeking responsible AI adoption.

## 1 Introduction

AI regulations are still in their infancy, with regions such as China and the European Union gradually regulating the AI landscape (Méndez-Suárez et al., 2023) (Table 1). Despite the rapid expansion of AI and the need for regulation, the United States still does not have an official AI framework (Binesh and Ghaharian, 2026; Ghaharian et al., 2025). While many industries have explored the ethics of AI, the hospitality and tourism industry is lagging despite its heavy reliance on human interaction (Binesh and Ghaharian, 2026). This scoping review addresses this gap by comprehensively analyzing the current literature on AI ethics in hospitality and tourism, and an in-depth analysis of the ethical dilemmas through the lenses of ethical theories such as epistemology and belief in AI. It also provides a risk assessment of AI systems in hospitality and tourism, offering actionable outcomes. Finally, we provide solutions for ethical concerns during the AI development cycle in a hospitality and tourism setting.

**Table 1:** Global approach to AI and data governance.

Aspect	GDPR dimension	AI Act	China’s AI regulations
Focus	Privacy and data protection	Risk-based AI regulation	State control and social values
Scope	Global (EU citizens’ data)	AI systems operating in the EU	Domestic, with government oversight
Transparency	Requires clear consent and disclosures	Mandates explainability of AI	Algorithm disclosures to users and state
Privacy	Data protection is paramount	Complementary to GDPR	Localized data storage required
Risk framework	Data-specific	Risk categories: high, limited, minimal	Avoids threats to social order
Ethical concerns	Protects individual autonomy	Focus on fairness and safety	Aligns with national cultural and ethical norms
Global applicability	Extraterritorial Scope	EU-centric, influences globally	Domestic data compliance

GDPR, General Data Protection Regulation (EU); AI Act, European Union Artificial Intelligence Act 2024 (European Union, 2024); extraterritorial scope, applies to organizations outside the EU that process EU citizens’ data.

This study contributes to ongoing research on AI ethics, particularly in the context of the hospitality and tourism industry. It enhances our understanding of how ethical frameworks, such as epistemology and belief theories, can be applied to the emerging challenges of AI in industries reliant on human interaction. Furthermore, this research contributes to the broader field of ethical AI development by expanding discussions on fairness, transparency, and accountability in AI systems designed for experience-based economies. By situating AI ethics within an industry that thrives on human connection, this study challenges traditional AI governance models and offers fresh insights into balancing automation with ethical responsibility in service-oriented businesses. The findings of this study can help researchers and practitioners alike maneuver the critical path of ethical AI.

Although AI ethics has been extensively discussed in domains such as healthcare, finance, and public governance, the hospitality and tourism literature remains fragmented. Existing studies have primarily examined customer acceptance of AI, service automation, digital transformation, and operational efficiency, while ethical concerns are often addressed as secondary issues. Furthermore, existing discussions tend to focus on individual ethical challenges such as privacy, bias, transparency, or trust rather than examining how these concerns collectively emerge from the ways AI systems generate, justify, and act upon knowledge claims about customers, employees, and destinations.

Consequently, three gaps remain. First, there is limited synthesis of AI ethics issues across hospitality and tourism sub-sectors. Second, literature lacks an integrative framework explaining how ethical concerns are connected rather than treating them as isolated phenomena. Third, few studies translate ethical concerns into operational governance mechanisms tailored to hospitality and tourism contexts.

To address these gaps, this review synthesizes the emerging literature on AI ethics in hospitality and tourism, develops an epistemological interpretation of ethical risks associated with AI-generated beliefs and inferences, and proposes sector-specific and lifecycle-based governance frameworks for responsible AI implementation.

Unlike prior reviews that focus on AI adoption, digital transformation, or general AI governance, this review specifically examines the ethical implications of AI deployment in hospitality and tourism. Rather than viewing privacy, bias, misinformation, transparency, autonomy, and accountability as separate concerns, the review interprets them through an epistemological lens. This perspective views AI systems as entities that generate beliefs about customers, destinations, employees, and operational environments, thereby linking technical performance with ethical responsibility. The review therefore contributes not only a thematic synthesis of ethical concerns but also a conceptual explanation of how those concerns emerge from AI-driven knowledge formation processes.

## 2 Methodology

This scoping review followed the PRISMA method to identify key ethical concerns, guidelines, barriers, and solutions associated with AI adoption within the hospitality and tourism industry (Boege et al., 2024; Page et al., 2021). The review was structured using an adapted PICOS framework, consistent with the population, concept, and context approach recommended for scoping reviews (Tricco et al., 2018). Eligible studies were those concerning hospitality and tourism stakeholders (customers, employees, managers, and tourists) affected by AI systems. The concept was AI applications, including generative AI, service robots, recommendation systems, chatbots, and biometric technologies. No comparator was required. Outcomes were ethical concerns, risks, guidelines, and proposed solutions. Eligible designs included peer-reviewed empirical and conceptual studies published in English between 2020 and 2025.

**Figure 1:**
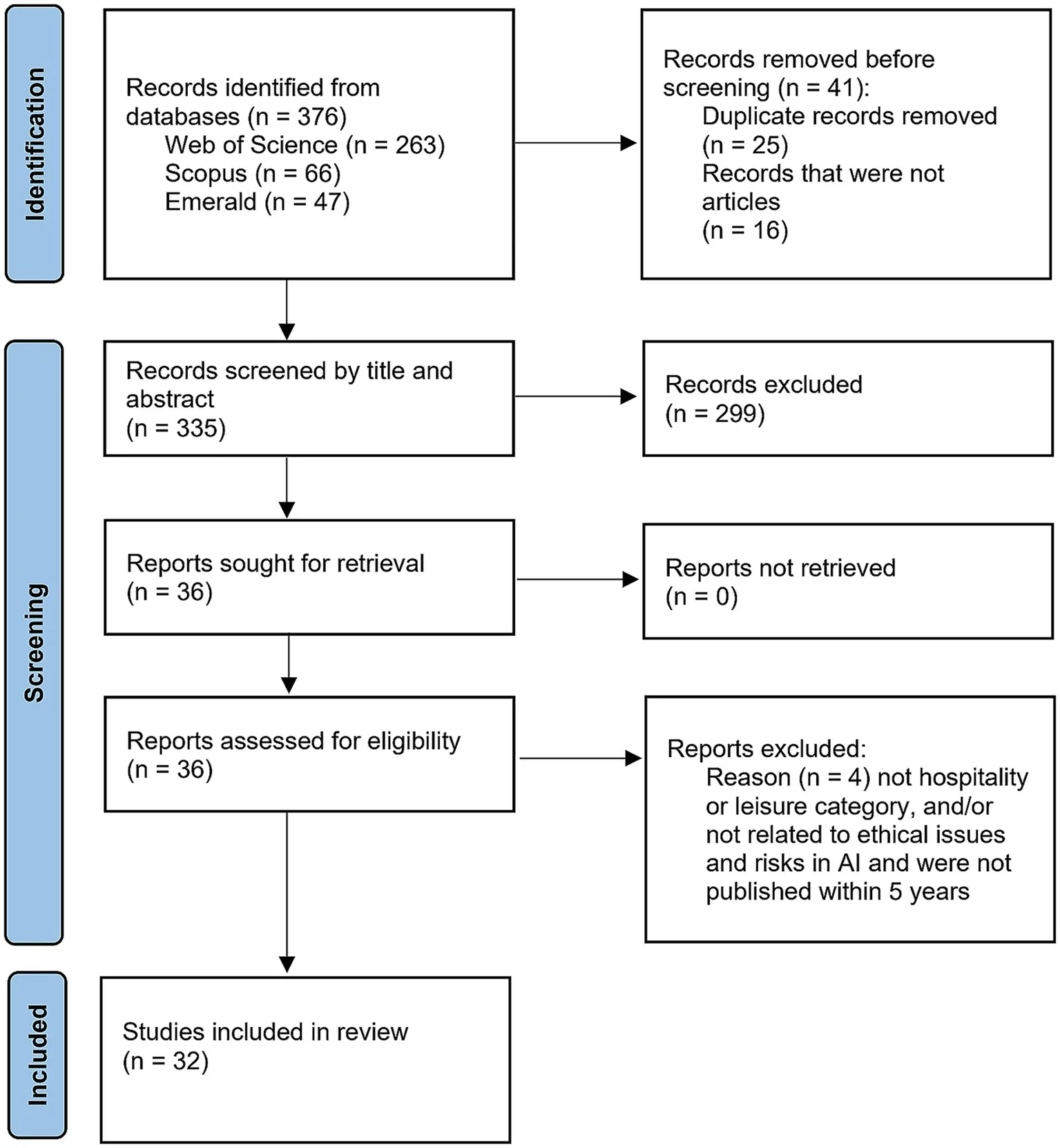
Flow diagram illustrating the systematic screening process for the AI ethics literature, showing the identification, inclusion, and exclusion stages that yielded 32 articles for analysis. Source: Page et al. (2021).

The literature search was conducted across Web of Science, Scopus, and Emerald in April 2025 using a systematic combination of five keyword groups. The full Boolean search strings for each database are provided in Appendix A.1 in Supplementary material. The search retrieved 376 records before deduplication. After screening (Figure 1), the final dataset included 32 articles on ethical issues and risks in AI within hospitality and tourism. All included articles were coded in Excel using inductive thematic analysis (Braun and Clarke, 2006). Both authors independently read the full texts and extracted statements describing ethical concerns, risks, guidelines, or solutions. Open codes were assigned to each statement, producing 16 sub-issues, which were clustered into nine broader themes through iterative comparison. Coding was performed independently by two researchers, and disagreements were resolved through discussion until consensus was reached. The nine themes were then mapped onto concepts from epistemology and the ethics of belief (for example, belief as information and doxastic wronging) by identifying the knowledge-formation failure underlying each theme, as summarized in Tables 2, 3.

Although critical appraisal is optional for scoping reviews under PRISMA-ScR (Tricco et al., 2018), we assessed study quality to contextualize the evidence. Empirical studies (n = 18) were appraised with the Mixed Methods Appraisal Tool (Hong et al., 2018), and conceptual papers (n = 14) were evaluated for theoretical grounding and argumentative coherence. Both authors appraised independently and resolved disagreements through discussion (Appendix A.11 in Supplementary material). No studies were excluded on quality grounds, consistent with the scoping purpose of this review. Themes supported mainly by conceptual work, such as autonomy and academic integrity, should be read as emerging rather than empirically consolidated.

## 3 Results

In our analysis of ethical issues and risks in AI-related studies, we identified 16 sub-issues grouped into nine broader themes. “Misinformation and Trust” was the most frequently discussed theme (n = 11), followed by “Bias and Discrimination” (n = 10) and “Transparency and Fairness” (n = 9). A complete list of the papers analyzed under each theme is provided in Appendices A.2 through A.10.

### 3.1 Misinformation and trust

Eleven articles cover this category and explore debates around (i) false visual data reproduction, (ii) generative AI risks in generating wrong information and academic plagiarism, and (iii) trust in human-robot interactions. Fernández Mateo (2022) and Crippen (2023) argued that the rise of AI has opened avenues for ultimate manipulation and fraud, especially fabricated images (e.g., in deep fakes). They urged that the advancement in AI-generated visualizations, if not well-regulated, will lead to fraud. From the end users’ perspective, Orr and Davis (2020) showed that according to twenty-one experts in AI, the ethical parameter of AI-generated data, fabrication of information, including biases, has evolved beyond AI design control. This is due to the nature of AI, which learns and grows as it is fed - which the systems somewhat ignore the reliability of the cumulative feeds prior to forming the algorithms (Orr and Davis, 2020; Roestamy et al., 2023; Zebrowski, 2021).

Sop and Kurçer (2024) simulated responses from ChatGPT on two different groups of 400 and 800 research respondents. In their study, they found that despite the ability of ChatGPT to generate statistical analysis, there are still questions around the quality and originality of ChatGPT’s output. This aligns with Dogru et al. (2024) and Nautiyal et al. (2023) regarding the inability of ChatGPT to disseminate accurate information for academic work in tourism and hospitality and other disciplines.

Bhaskar and Sharma (2022) discussed ethical considerations related to AI and robotics in the tourism and hospitality industry, including issues such as privacy, prejudice, and ethics. They argued that the drawbacks of AI adoption in the travel and hospitality industry include restricting customer autonomy and making sense of AI video surveillance as a threat to customers. They addressed the spillover effect of wrongly used video surveillance that triggers data leakage, privacy breaches, and customer mistrust. Likewise, Hsu et al. (2024) focused on proposing reliable AI-generated content and recommendations for tourists. They argued that tourist experience and trust from using AI could be mitigated by leveraging fine-tuning large language models with input from high-quality datasets across multiple stakeholders.

### 3.2 Bias and discrimination

Ten articles were focused on subtopics: (i) Bias in AI-generated content, (ii) AI Governance, and (iii) AI and inequalities.

Researchers agree that the complex nature of AI ethical perceptions may lead to inevitable bias (e.g., Lin et al., 2024; Orr and Davis, 2020). AI system design is not a consensus product between the target audience and the firm (Orr and Davis, 2020). Additionally, the decision in AI design may exclude the dataset from underrepresented samples, making bias more vulnerable (Mamychev et al., 2021). Orr and Davis (2020) highlighted an imbalanced condition between decision power and technical expertise. At the same time, Azzutti et al. (2021) and Lin et al. (2024) agreed that AI’s potential bias can be due to inherent data limitations. For example, AI is still prone to representation issues where the data does not represent equal demographic distribution and marginalized groups, creating bias (Azzutti et al., 2021; Chen et al., 2024). They agreed on one argument: excluding the general audience (or the experts) in the first phase of AI system development creates bias. Different types of bias were explored, such as data and trading algorithm bias (Azzutti et al., 2021), cognitive, intervention, and feature selection and recommendation bias (Zebrowski, 2021), perceived feedback loop bias, model development bias, and cultural and societal bias (Lin et al., 2024). Additionally, Dogru et al. (2024) reported possible legal issues in using generative AI, such as bias, privacy, and transparency. These problems may reflect unfair treatment of race, ethnicity, or people with disabilities, data leakage, and bias in the rationale behind generative artificial intelligence recommendations (Dogru et al., 2024).

The second sub-topic synthesizes how governance theories have been utilized to regulate and inform bias and discrimination of AI-generated technologies, which falls under policy or regulatory research. Auld et al. (2022) explained the extent of governing ethical standards for AI and proposed three key pathways for governing AI ethics. There are opposing and defending, where private entities challenge government interventions and regulations, often resorting to private governance mechanisms to oppose government standards. Engage and push, where private entities collaborate with public organizations to advance AI ethical standards. However, this approach is unstable due to changing governance requirements and criticisms of existing regulatory initiatives. Auld et al. (2022) propose the following to remove bias and inequality in AI: (i) AI for inclusive governance with focus on marginalized groups and vulnerable populations, (ii) ethical guidelines and standards to mitigate harm and uphold fairness, (iii) regulatory measures on transparency and accountability, including audience consent on automated decision-making, and (iv) a community-centric approach that focuses on indigenous data sovereignty for equitable and equal AI system outreach.

The third sub-topic focuses on inequalities amplified by AI and its governance. Derave et al. (2022) uncovered the issues of discrimination towards third-world travelers in European borders. Border AI-enabled security systems such as facial recognition, physical traits, and risk profiling can pose data biases and may result in discriminatory treatment of travelers, especially those from the Third World. Méndez-Suárez et al. (2023) analyzed the effectiveness of GDPR against firms’ AI malpractice, especially in customer data breaches. According to Méndez-Suárez et al. (2023), manipulating users’ privacy in digital marketing has sparked issues within firms’ AI malpractices. Manjarrés et al. (2021) emphasized that AI inequalities stem from unequal access to technology. Their thesis stresses the social issue that underprivileged economic groups may not have similar access to technology as the affluent. Without proper governance, the underrepresented communities may be at higher risk of data privacy breaches.

### 3.3 Transparency and fairness

Nine articles under three different sub-topics; (i) Major themes of transparency and fairness, (ii) future technologies for sustainability and ethical standards, (iii) transparency and fairness for risk mitigation and safety.

Lin et al. (2024) gathered customers’ perceptions through case studies on AI adoption and autonomous service robot interactions. Their study opened the role of AI and human-robot interaction problems such as the uneasy feeling of robot surveillance, the fear of privacy infringement, malicious use of customer data without transparency, unreliable service support, and biased robot features. Furthermore, Chon and Hao (2025) proposed a bigger idea in technological advancement for the travel, tourism, and hospitality sectors. They highlighted that advancement in AI may affect the ethical ramifications of the industry and its externalities.

Manjarrés et al. (2021), on AI for a fair, just, and equitable world, explained the logic from the externality that ethical issues may extend to a broader social and environmental impact. For example, suppose the ethical advancement of AI in travel, tourism, and hospitality activities does not encourage green practices, such as not visiting overcrowded sites, preventing racial discrimination, or intervening in food waste habits. In that case, the effects may outweigh the ethical dilemma alone (Manjarrés et al., 2021). Hence, Chon and Hao (2025) proposed accountability for the AI process. This process would enable constant disclosure and trustworthy recommendations on all AI operations devices in offering automation services that accommodate all activities of tourists (e.g., museum chatbots, hotel check-in kiosks, travel maps, local food and lodge reviews, etc.). Additionally, from a technological point of view, Hadjistavropoulos (2023) noted the importance of adaptation in how practitioners treat technology that adheres to ethical principles. Hadjistavropoulos (2023) described how much advancement in AI has occurred in healthcare services. Rather than proposing the AI design to address ethical concerns, he proposed human adaptation to ever-changing interactive healthcare technologies while appreciating the utmost ethical standards.

The discussions on risk mitigation and safety are also central to AI-generated technologies’ transparency and fairness efforts (Méndez-Suárez et al., 2023; Vidgen et al., 2020). Oravec (2021) emphasized the importance of risk prevention in human versus AI/automation interactions. Coming from two studies where Oravec delved into industrial robot risks, while Bickley and Torgler (2023) on cognitive architecture of AI ethics, both researchers urged risk mitigation promotion, awareness, and training on AI and automation. While both researchers are nothing against the ease of automation, they stressed the dangers of the undisclosed risks of AI and automation. Oravec (2021) clearly stated the word “death by robot” to advocate harm transparency within the workplaces that employ robots and automation infrastructures. Oravec (2021) belief in the coexistence of humans and robots does not hinder him from campaigning risk outlook and prevention. Meanwhile, Bickley and Torgler (2023)‘s notion of the “black box” of AI must have demanded individuals to question what is beyond the design of AI systems’ cognitive architecture. This part of making individuals aware of their predetermined decisions made by AI must be deemed as an ethical requirement (Bickley and Torgler, 2023). The AI must possess transparency, explainability, and accountability (Bickley and Torgler, 2023). This is especially critical when the AI function works in helping the human decision during a crisis (e.g., health decisions, well-being, finance, law).

### 3.4 Privacy and data management

Seven studies fall under four themes: (i) privacy, data use, and information accuracy issues in marketing and decision-making processes, (ii) legal concerns related to intellectual property use, (iii) legal standards, and (iv) discrimination and human rights.

Oravec (2021) explored ethical aspects of AI and found that generative AI, machine learning, and automatic customer services such as robots, kiosks or chatbots pose a high risk of privacy breaches and information accuracy, a concern echoed in broader research on human trust in AI advice (Vodrahalli et al., 2022). Similarly, Hsu et al. (2024) elaborated an AI design blueprint that must cater to improved database handling and lack of recommendation bias. Bulchand-Gidumal et al. (2024) conducted a grounded theory study involving AI experts and hospitality marketing professionals. Through interviews and focus groups, they identified key themes illustrating how AI transforms hotel marketing by enhancing personalization, optimizing organizational processes, supporting employees, and introducing challenges related to data ethics, sustainability, and return on investment.

In the regulatory area, Vidgen et al. (2020) recommended solutions for data management risks such as security breaches and unauthorized access to customer profiling through the General Data Protection Regulation (GDPR). They proposed data management cores around lawfulness, fairness, transparency, and data accuracy. Their proposal also directed policy focus on individuals’ rights - specifically the right to access their data, right to rectification, right to erasure, right to data portability, and right to object to the use of their data for marketing purposes. Similarly, Méndez-Suárez et al. (2023) focused on the European Union AI policy setting by examining 31 annotated observations of publicly traded companies fined for severe AI violations. Their findings show that most European companies committed privacy violations by manipulating digital marketing information.

Under the fourth category - discrimination and human rights, Derave et al. (2022) looked at the case of the European Travel Information and Authorization Systems (ETIAS) and the EU information technology system. They reported that the utilization of AI in protecting privacy and data management is also geared towards overseeing discriminatory practices, particularly when travelers from third-world countries visit the EU.

### 3.5 Impact on society

The impact of AI on society has been explored through eight articles on (i) impact on humans such as employee replacement, (ii) legal and social impacts and (iii) behavioral identity and culture impacts. The advancement in AI technologies is not without consequences. With AI taking over areas of the customer service process - such as automatic check-in and check-out, seamless reservations, and automated room service - the threat of human job replacement is non-negotiable (Lin et al., 2024). Kim et al. (2023) emphasized that the adoption of AI must be balanced with reformative policy adjustments to protect the fate of the human workforce. Similarly, Shklovski and Némethy (2023) raised concerns that AI can create inequality in society, particularly through an overemphasis on engineering skills that may widen gaps in economic power. This means those who cannot catch up will struggle to survive the disruption. Arvan (2023) further argued that AI threatens academia by enabling ideation and writing.

While AI advancement can empower those with skills, education, and economic power, it often leaves marginalized groups behind, creating problems related to justice and fairness (Kim et al., 2023). With AI’s presence in the workplace, employees have gradually become accustomed to robot interactions - mirroring the experience of customers in automated service encounters. This shift in human-to-AI interaction has eroded emotional connections between individuals. AI replaces both technical processes and the human intensity involved in communication and problem-solving, including emotional attachment. As a result, emotional alienation becomes inevitable, with a reduction in empathy and engagement. Another side effect is emotional labor, which affects human attentiveness and attention to detail as AI increasingly handles tasks. Within hospitality, AI in service operations may manipulate authentic human emotions. If the distribution of work between AI and humans is not governed equitably, emotional attachment and human service are at risk of being replaced (Sheng and Wang, 2022).

On the positive side, Kim et al. (2023) found that AI could enhance public infrastructure such as smart cities, more reliable public transport, and improved public services. Mamychev et al. (2021) explored multiple angles of AI’s societal impact, noting that AI can support human functions in decision-making and labor, enhance data-driven judgments in business or policy, and even contribute to the arts and creative industries. Nevertheless, scholars agree that the ethical dilemmas surrounding AI are far from resolved especially when it comes to the reconstruction of social hierarchies and technocratic governance. As decision-making and judgment shift toward AI-driven systems, there is a growing risk that important decisions (e.g., education, social, or cultural preservation policies) may fail to account for minorities, further deepening social stratification (Jaillant and Caputo, 2022; Mamychev et al., 2021).

### 3.6 Ethics in AI development

The eight papers covered: (i) ethical privacy design, (ii) ethical fairness and bias design, and (iii) ethical governance.

Scholars agree on the importance of user privacy in AI system development (Bulchand-Gidumal et al., 2024; Chen et al., 2024; Clayton and Sibbald, 2020). This is particularly important due to the inherent nature of AI, which involves autonomous decision-making and personalization. Lack of transparency can lead to misuse of user profiles and surveillance data.

Secondly, the discussion on developing ethical guidelines for AI adoption also embraces the area of AI fairness and bias. For instance, there is a need for policies that ensure equal access to AI in sectors such as education or healthcare (Clayton and Sibbald, 2020), which may help address the economic gap between the wealthy and the low middle-class society. This policy emphasis cannot be separated from the challenges of AI-bias design, which does not yet adequately cater to a broader social stratum (Bickley and Torgler, 2023; Bulchand-Gidumal et al., 2024; Manjarrés et al., 2021).

Aside from business needs, AI development research must be targeted to refine socio-economic activity aspects where AI adoption is mainly facilitated. Jaillant and Caputo (2022) and Mamychev et al. (2021) argued that AI may not fully capture a fair and diverse algorithm. Jallian and Caputo specifically called out the importance of AI fairness toward all audiences of digital historical databases, warning of questionable AI biases that may lead to favoring a particular culture or group of people.

One route to addressing such design-stage bias is participatory design, which involves stakeholders directly in system development and has long been used to embed diverse perspectives into technology design (Bellandi et al., 2012). Extending this approach to AI development in hospitality could help surface the needs of underrepresented guest and employee groups before biased assumptions become encoded in deployed systems.

From the companies’ perspective, Vidgen et al. (2020) proposed themes for an ethical agenda in firm AI adoption and stakeholder management. Their thesis argued that to maintain a balanced value creation and ethical responsibility, firms must uphold the ethical principles of utility, right, justice, common good, and virtue. Vidgen et al. emphasized that ethics should never be separated from the conceptual plan of AI adoption. Ethics is the core design for value creation. Finally, Auld et al. (2022) also brought up ethical governance. They stress that governments should regulate AI, and AI systems should be constantly reviewed by internal governing experts. The risks to public audiences resulting from AI are human rights issues - hence requiring government intervention.

### 3.7 Security and safety

Six articles, with primary debates falling under (i) safety issues in human-robot collaboration in daily operations, (ii) AI adoption in public spaces (e.g., destinations, parks) and its implications for public policy, and (iii) protocols for privacy and human protection.

Studies by Kim et al. (2023) and Lin et al., 2024 highlight safety concerns from different contexts. Kim et al. (2023) argue that autonomous service robots may pose physical dangers during human interaction, while Lin et al. (2024) warn that ChatGPT risks uninformed data collection and third-party sharing. Both emphasize cybersecurity threats. Service robots connected to the Internet of Things (IoT) make personal information vulnerable to hacking (Lin et al., 2024), and ChatGPT may offer unsafe or biased travel advice due to flawed algorithms (Kim et al., 2023).

Oravec (2021) echoed Kim et al. (2023)’s concerns by examining industrial robots and their potential to harm humans both physically and ethically. He argued that privacy risks and surveillance-related uncertainty can create mistrust in automation. His study identified three consequences of workplace incidents involving robots: (i) fear of future robot interaction, (ii) mistrust, and (iii) anti-robot sentiments. While Oravec suggested that promotional robot designs may attract user attention, he cautioned that such appeal does not ensure human safety without a focus on protective design.

Kim et al. (2023) and Méndez-Suárez et al. (2023) also discussed AI’s impact on individuals’ perceived safety and behavioral responses. They found that AI safety designs influence tourist and community satisfaction, especially when combined with visible safety cues, emotional reassurance, and user confidence. Across disciplines, scholars recommend integrating safety technologies into policy frameworks through stakeholder collaboration.

Roestamy et al. (2023) conducted a bibliometric analysis of legal issues related to AI in tourism and emphasized the importance of data privacy, accountability, and well-communicated regulations. Similarly, Fernández Mateo (2022) advocated stronger privacy protection. Both studies support advancing digital governance and enacting robust data security policies to safeguard users’ rights.

### 3.8 Autonomy and responsibility

This category contains three articles: (i) defining human autonomy threats in AI adoption and (ii) reducing AI algorithms’ bias with human-in-the-loop strategies. Bhaskar and Sharma (2022) and both discussed ethical concerns associated with AI, although from different perspectives. While Bhaskar and Sharma reviewed the broader implications of AI adoption in tourism and hospitality, including issues related to privacy and ethics, Fernández Mateo (2022) specifically examined the implications of AI for individual autonomy. Conversely, Fernandez-Mateo centered his focus on the lack of transparency and the importance of informed consent. He argued that despite AI’s ability to streamline services, its opaque operations and underlying algorithm have, unfortunately, jeopardized individuals’ authority and decision-making.

Morosan and Dursun-Cengizci (2024) assert that the diminishing of human control is an inevitable consequence of using AI. They explain the “off-the-loop” condition as one with potentially limited power to override or interfere with AI decisions. This effect can be exacerbated by the inherent unpredictability and lack of transparency in AI decision-making tools; commonly referred to as the black box effect. Fernández Mateo also mentioned that when both firms and customers are not in a position to raise concerns and advocate for human oversight, human decisions will soon be overtaken by algorithmic ones. They emphasized that limiting AI autonomy is the only way to mitigate this risk.

### 3.9 Academic integrity

Three articles were examined: (i) AI-generated plagiarism, (ii) AI for academic publishing review, and (iii) the shift in academic philosophy.

Nautiyal et al. (2023) brought escalating problems in AI-generated plagiarism for academic writing. The central argument on ChatGPT is that it can generate trustworthy recommendations that blur academic authorship’s accountability. They also raised concerns about the behavioral effects of ChatGPT on students, as they argue that ChatGPT can create students’ heavy reliance on AI-generated ideas, hence creating the erosion of creativity and critical thinking. Using AI tools for research communications, including peer review and publication, also calls for oversight. Similarly, Dogru et al. (2024) call publishers to use robust AI detection software. They believe that the common plagiarism in research publications occurs due to a lack of oversight from editorial members. They also commented on possible bias in the peer review process with AI. They agreed that AI can streamline the communication process during peer review, highlighting the need for human intelligence (Dogru et al., 2024; Nautiyal et al., 2023).

Arvan (2023) focused on the operational aspect of AI in academia. Arvan mentions the paradigm shift and argues that using AI to support academic work creates broader ethical dilemmas, such as intellectual property rights and research originality. He also contends that epistemological erosion may occur as scholars use AI to review manuscripts (Arvan, 2023). All authors agreed on the need to regulate AI, oversee it, and ensure human involvement.

Based on this comprehensive search of the literature, we created a list of possible future research questions in each category (Table 4).

**Table 4:** Future research based on the scoping review themes.

Theme	Future research
Privacy and Data Management	(i) How can bias mitigation framework be examined to prevent discriminatory outcomes of AI systems? (ii) How can AI marketing be designed to safeguard customer privacy and build trust? (iii) Exploratory research on public trust factors for AI surveillance in the public sphere (iv) How can experimental studies with AI-generated design prototypes for decision-making address data risk issues?
Security and safety	(i) How can an exploratory study (e.g., expert opinions) identify automation safety optimization in the workplace (ii) How do destination management offices/hospitality businesses mitigate vulnerabilities in AI-enabled services (e.g., hacking risk, tourist mobility surveillance risks, etc.) (iii) To what extent can the psychological aspects of tourists be tested for AI-driven safety measures in a destination or hotel? (iv) How can case studies in travel and tourism risks be leveraged to underpin informed policy studies?
Transparency and fairness	(i) To what extent are sensitive operations such as customer recommendations developed to meet transparency and explainability standards? (ii) How can government, tourism or hospitality firms enable fair AI-enabled design catering to a diverse market background? (iii) What role does the transparency of cognitive architecture play in reducing mistrust and promoting ethical AI adoption?
Autonomy and responsibility	(i) How do psychological and sociological factors affect perceived AI risk acceptance or resistance? (ii) Qualitative research on guiding rules for AI autonomy in human decision-making (iii) Case study research on benchmark policies and regulations for AI autonomy and responsibility
Bias and discrimination	(i) How can the participatory design framework (e.g., stakeholder engagement) bridge AI bias issues for underrepresented groups? (ii) To what extent can governance theory be leveraged to promote fairness and discrimination in healthcare, travel, and service sectors? (iii) How can ethics and public policy intersect to answer pressing issues in AI adoption for public goods and equality
Ethics in AI development	(i) How can user-controlled privacy mechanisms be designed to protect customers from misuse of surveillance data during autonomous decision-making? (ii) How can firms operationalize ethical principles such as justice, common good, and virtue in their AI adoption frameworks to balance business value creation with ethical responsibilities?
Impact on society	(i) How can policymakers and businesses create strategies to mitigate AI-related job displacement in customer service and operations, ensuring fair transitions and upskilling for the workforce? (ii) What are the long-term effects of AI-driven emotional alienation on workplace dynamics, customer satisfaction, and human empathy in service industries? (iii) How can AI take-over affect employee turnover and reduce customer loyalty, and profitability?
Misinformation and trust	(i) How can stakeholders/expert research on AI adoption inform the user and regulators on fraudulent image or data manipulations in AI tools? (ii) To what extent does scholars’ point of view research navigate the use of AI for academic standards and ethics (iii) To what extent does tourism research on AI surveillance’s perceived safety and trust inform destination managers and policy makers? (iv) How to develop machine learning models to enhance trust and maintain individuals’ autonomy for AI-generated recommendations?
Academic integrity	(i) How can educational institutions establish oversight mechanisms to balance the benefits of AI tools with the need to maintain academic integrity and originality? (ii) What are the long-term effects of AI-generated content on the practices of academic research and the development of scholarly knowledge?

## 4 Discussion

Before examining the findings through an epistemological lens, it is important to clarify the relevance of epistemology to AI ethics. AI systems continuously generate knowledge claims about customers, destinations, employees, and operational environments through prediction, classification, and recommendation processes. Consequently, many ethical concerns associated with AI can be understood as epistemological challenges involving the quality, reliability, transparency, and justification of AI-generated beliefs.

### 4.1 The epistemology of AI in hospitality and tourism

Epistemology is concerned with nature, justification, reliability, and limits of knowledge. Its relevance to AI ethics stems from the fact that AI systems continuously generate knowledge claims about users, environments, and future actions. In hospitality and tourism, AI systems infer customer preferences, predict behaviors, classify risks, recommend destinations, determine prices, and personalize services. Ethical concerns arise when these inferences are inaccurate, biased, insufficiently justified, or impossible to scrutinize. Consequently, many ethical issues identified in this review can be understood as epistemic failures that subsequently produce ethical harm.

Viewed collectively, the nine themes identified in this review are not independent ethical issues but manifestations of broader epistemological challenges. Bias reflects distorted knowledge generation, misinformation reflects inaccurate knowledge claims, opacity reflects hidden justification processes, and privacy concerns reflect excessive knowledge extraction. This perspective provides a unifying explanation for the ethical challenges associated with AI deployment in hospitality and tourism.

### 4.2 The ethics of belief for AI in hospitality and tourism

In addition to the epistemology of AI, we looked at the findings of this review through the lens of ethics of belief for AI (Table 2). Three concepts from the ethics of belief guide this analysis. Doxastic wronging occurs when an agent wrongs a person simply by holding an unjustified or demeaning belief about them, before any action is taken (Basu and Schroeder, 2019). Morally owed beliefs are beliefs an agent is morally required to hold or avoid, given the stakes for others. Pragmatic and moral encroachment is the view that the practical or moral consequences of being wrong raise the amount of evidence needed before a belief is justified.

**Table 2:** Epistemological interpretation of AI ethics themes in hospitality and tourism.

Theme	Epistemological problem	Ethical outcome
Misinformation and trust	False knowledge claims	Deception
Bias and discrimination	Distorted knowledge formation	Unfair treatment
Transparency and fairness	Hidden justification	Lack of accountability
Privacy and data management	Excessive knowledge extraction	Surveillance
Impact on society	Displacement of human knowledge and judgment	Job loss and social alienation
Ethics in AI development	Unvalidated knowledge embedded in the design stage	Encoded injustice
Security and Safety	Unreliable knowledge systems	Harm
Autonomy and responsibility	AI overriding human judgment	Loss of agency
Academic Integrity	Asserting unverified machine-generated claims as knowledge	Erosion of scholarly trust

Epistemological problems describe failures in how AI systems form, justify, or assert knowledge; ethical outcomes describe the resulting harm to stakeholders.

**Table 3:** Mapping findings based on the ethics of belief for AI in hospitality and tourism.

Logic-based approach to belief	Applications for hospitality and tourism	Ethical concerns	Related themes	Proposed solutions
Belief as information	AI travel recommendation systems; dynamic pricing	Biased suggestions manipulating users; opaque decisions limiting autonomy; privacy risks from surveillance	Bias and Discrimination; Transparency and Fairness; Autonomy and Responsibility; Privacy and Data Management	Enhancing transparency; enforce data privacy laws; require explicit user consent
Tractable logic approach	Dynamic hotel pricing; service robots for check-in, room service	Price discrimination based on digital footprint; cultural insensitivity; rigid logic ignoring human context	Bias and Discrimination; Privacy and Data Management; Impact on Society	Introduce fairness checks; improve explainability; embed ethics in design constraints
Belief as Rapid Discovery	AI-generated tourism content; sentiment analysis; deepfakes	Misinformation in marketing; emotional manipulation; systemic discrimination in rapid decisions	Misinformation and Trust; Security and Safety; Bias and Discrimination; Academic Integrity	Implement fact checking; set emotion AI boundaries; establish governance for real-time AI

Belief as information, treating any data an AI system holds as its beliefs; tractable logic approach, belief formation limited to inferences computable within feasible time and resource constraints; belief as rapid discovery, beliefs generated through fast, large-scale pattern detection.

**Table 5:** AI risks and regulatory landscape in hospitality and tourism.

Sector	Risk level	Risk criterion	Example	Regulatory implication
Accommodation	Unacceptable	Manipulates consumer behavior or undermines autonomy	AI manipulates guest behavior to maximize spending without transparency	Prohibited due to ethical violations
	High	Using biometric data significantly affects individual rights	Biometric room entry and identity verification	Requires fairness testing, security safeguards, and human oversight
	Limited	Influences customer decisions but does not determine outcomes	AI-generated reviews based on previous guest feedback	Transparency and disclosure requirements
	Minimal	Provides operational support with limited ethical impact	Chatbots providing basic customer assistance	Users must be informed they are interacting with AI
Food and beverage	Unacceptable	Produces discriminatory outcomes	AI algorithms adjust prices based on ethnicity or socioeconomic status	Prohibited outright to prevent exploitation
	High	Uses biometric or sensitive personal data	Facial recognition systems used to identify customers and personalize services	Strict compliance with data protection and consent requirements
	Limited	Personalizes recommendations based on user data	Menu recommendation systems based on previous orders	Disclosure of data use and recommendation logic required
	Minimal	Automates routine administrative functions	AI for table reservations and queue management	Standard transparency requirements
Travel and transportation	Unacceptable	Violates fundamental rights through profiling	AI-enabled travel systems scoring passengers based on political or ideological views	Prohibited under AI governance frameworks
	High	Using biometrics or affects mobility and security decisions	Biometric screening at airports	Human oversight, conformity assessments, and privacy safeguards required
	Limited	Travel decisions influences without determining outcomes	AI-based route optimization and travel recommendations	Users informed about data use and recommendation processes
	Minimal	Provides informational support	Automated travel information and schedule updates	Transparency encouraged
Tourism and recreation	Unacceptable	Manipulates consumer behavior through exploitative recommendations	AI recommending overpriced activities based on urgency or vulnerability	Prohibited due to unethical manipulation
	High	Uses biometric or emotional recognition technologies	Virtual tour guides using facial recognition to assess visitor emotions	Explicit consent and ethical safeguards required
	Limited	Personalizes visitor experiences	AI-based itinerary planning and activity recommendations	Transparency regarding recommendation criteria required
	Minimal	Provides basic informational assistance	Automated information kiosks at attractions	Notification that AI is being used
MICE	Unacceptable	Creates discriminatory treatment of participants	AI assigning different service levels based on attendee profiles	Prohibited due to unfair treatment
	High	Uses biometric monitoring or behavioral tracking	Facial recognition for attendee tracking and crowd analysis	Human oversight and periodic audits required
	Limited	Supports planning and allocation decisions	Recommendation systems for event layouts or seating arrangements	Disclosure of automated decision support required
	Minimal	Automates scheduling and coordination tasks	Automated scheduling tools for event planners	Standard transparency requirements
Spa and Wellness	Unacceptable	Manipulates health-related decisions or exploits vulnerable individuals	AI recommending unnecessary treatments based on manipulated health data	Prohibited due to ethical and health concerns
	High	Influences health or wellness decisions	AI health-monitoring systems recommending wellness routines	Compliance with health and safety standards required
	Limited	Personalized services using customer data	AI personalization of spa services and wellness programs	Notification of data use and personalization required
	Minimal	Provides administrative support	AI-powered appointment scheduling	Basic transparency requirements
Entertainment	Unacceptable	Deliberately deceives users or misrepresents content	AI-generated content falsely representing cultural performances as authentic	Prohibited due to deception and misinformation
	High	Influences crowd safety or security	AI-driven crowd management systems at events	Safety testing and conformity assessments required
	Limited	Influences entertainment choices	Recommendation engines suggesting performances or activities	Transparency regarding recommendation logic required
	Minimal	Supports routine event administration	AI systems generating digital tickets and access passes	No significant additional requirements beyond transparency

MICE, Meetings, Incentives, Conferences, and Exhibitions. Risk tiers (unacceptable, high, limited, minimal) are adapted from the four-level classification of the European Union AI Act.

**Table 6:** AI life cycle risks and solutions.

AI life cycle stage	Epistemological concerns	Ethical belief concerns	Proposed solutions
Problem definition	Defining scope, truth, and goals	Identifying beliefs	Clarify whether AI should prioritize guest satisfaction, revenue, sustainability, or a balanced approach
Data collection	Truth, data reliability	Avoiding epistemic injustice	Use diverse datasets to reduce bias and ensure fair representation
Model development	Justification of outputs	Morally owed beliefs	Design models that honor cultural, personal, and accessibility preferences of guests
Deployment	Transparency, reliability	Doxastic wronging	Clearly explain chatbot outputs and prevent misleading or discriminatory recommendations
Feedback and improvement	Adaptability, evidence use	Pragmatic/moral encroachment	Continuously refining AI using guest feedback to enhance fairness and inclusivity

Doxastic wronging, wronging a person by holding an unjustified belief about them; morally owed beliefs, beliefs an agent is morally required to hold or avoid given the stakes for others; pragmatic/moral encroachment, the view that practical or moral stakes raise the evidence required to justify a belief; epistemic injustice, wronging individuals in their capacity as knowers, for example when their data is misrepresented.

Doxastic wronging emerges as a recurring theme, where AI holds and acts upon unjustified beliefs. For example, biased AI booking algorithms can lead to discrimination by prioritizing wealthier guests. The notion of morally owed beliefs is also highly relevant. AI in hospitality and tourism has a moral obligation to ensure fairness, inclusivity, and accurate representation of travel conditions. Cases where AI recommends unsafe destinations due to outdated or incorrect data show the importance of constantly validating these algorithms. In this context, pragmatic and moral encroachment becomes a crucial factor; AI algorithms should not only focus on algorithmic efficiency, but they should also prioritize guest autonomy, safety, and privacy. AI injustice keeps repeating under this theory as well.

#### 4.2.1 Belief as information in AI-driven hospitality and tourism

AI systems in hospitality and tourism operate under the premise that belief is an accumulation of structured, stored, and retrieved information to inform decision-making processes. The ethical concerns surrounding AI adoption in the industry stem from how these systems gather and process data, often reinforcing biases and moral dilemmas. For instance, Lin et al. (2024) and Méndez-Suárez et al. (2023) highlight AI’s role in influencing consumer choices through personalized recommendations and algorithmic curation, which raises ethical concerns regarding data transparency and manipulation. AI-driven recommendation systems, such as those used in travel booking platforms and dynamic pricing models, function based on historical user behavior but may also introduce algorithmic biases (Dogru et al., 2024). The perceived reliability of these AI models is tied directly to how belief, as information, is encoded and used. A major ethical dilemma emerges when AI lacks explainability in decision-making, particularly in customer interactions. Oravec (2021) warns of the opacity in AI’s cognitive processes, arguing that users lack insight into why AI suggests certain services or travel destinations over others. This concern extends to AI surveillance in tourism spaces, where privacy risks emerge as AI collects vast amounts of user data without explicit consent (Kim et al., 2023).

Viewed through the lens of belief as information, several ethical themes identified in this review can be understood as failures in AI knowledge formation. Bias and discrimination emerge when the information used to construct AI beliefs is incomplete or systematically skewed, while misinformation and trust issues arise when AI-generated outputs are treated as reliable knowledge despite being based on inaccurate or insufficient data. Privacy concerns reflect the ethical implications of excessive information extraction, whereas transparency concerns stem from users’ inability to understand how AI systems transform information into recommendations and decisions. Consequently, the ethical challenges surrounding AI in hospitality and tourism are not isolated problems but interconnected outcomes of how AI systems collect, process, and act upon information about customers, employees, and destinations.

#### 4.2.2 The tractable logic approach to AI belief in hospitality and tourism

The tractable logic approach holds that an agent’s beliefs are limited to what can be logically derived within feasible time and computational constraints, rather than everything its information implies. AI-driven hospitality and tourism applications adhere to this principle by simplifying decision-making processes, albeit at the cost of nuanced ethical considerations.

For instance, dynamic pricing algorithms in hotel revenue management rely on predictive modeling and heuristic-driven logic, which often exclude non-quantifiable factors such as consumer fairness and ethical concerns (Dogru et al., 2024). While tractability ensures real-time pricing optimization, it also limits ethical oversight, as AI cannot fully consider the subjective experiences of diverse travelers. Kim et al. (2023) discuss how AI-driven pricing models often lead to discriminatory pricing strategies, disproportionately affecting customers based on their digital footprints rather than their preferences.

Similarly, AI-enabled service robots operate on predefined logical constraints, determining their ability to interact with customers. Morosan and Dursun-Cengizci (2024) discuss how robots in hospitality environments follow tractable logic models, enabling efficiency in check-in processes, room service delivery, and concierge functions. However, the inflexibility of these systems introduces ethical risks, particularly when robots fail to account for cultural sensitivities, accessibility concerns, or emotional nuances in guest interactions.

Additionally, the black-box nature of AI decision-making introduces challenges in auditability and accountability. As Bickley and Torgler (2023) note, AI-driven decision logic may function within predefined constraints, yet these constraints often fail to accommodate real-world ethical complexities, such as worker displacement, privacy infringements, and customer manipulation. The limitations of the tractable logic approach suggest that while AI can efficiently process structured belief systems, it fails to incorporate broader ethical contexts essential for fair and transparent AI integration in hospitality and tourism.

Through the tractable logic perspective, the ethical concerns identified in this review arise when complex human situations are reduced to computationally efficient decisions. This helps explain issues related to fairness, autonomy, and responsibility, where optimization objectives may outweigh contextual human judgment. Consequently, tractable logic highlights the ethical trade-offs between operational efficiency and human-centered service in hospitality and tourism.

#### 4.2.3 Belief as what one could rapidly discover: AI’s ethical adaptability

The notion that belief encompasses what AI can rapidly infer underpins the adaptive capabilities of AI in hospitality and tourism. AI models are designed to process real-time data, identify patterns, and make immediate inferences based on available information. However, this rapid discovery mechanism raises significant ethical concerns regarding misinformation, surveillance, and decision autonomy.

For instance, AI-generated misinformation in tourism marketing, whether through misleading chatbot recommendations or deepfake travel content exemplifies the risk of AI’s rapid inference mechanisms (Fernández Mateo, 2022; Sop and Kurçer, 2024). AI systems synthesize data from various sources, which may generate inaccurate or biased insights, impacting traveler trust and ethical responsibility. Hsu et al. (2024) argue that AI’s ability to generate content must be counterbalanced with robust verification frameworks, ensuring that recommendations align with ethical and factual accuracy.

Furthermore, automating decision-making processes in AI-driven tourism applications amplifies ethical concerns about personal autonomy. Bhaskar and Sharma (2022) cautioned that the increasing use of AI in tourism and hospitality raises ethical concerns, particularly regarding privacy, prejudice, and the responsible use of customer data, as AI systems become more integrated into travel planning and service delivery. Similarly, Lin et al. (2024) highlights that AI-driven recommendation systems in smart tourism may reinforce digital filter bubbles, limiting travelers’ exposure to diverse experiences.

From an operational standpoint, AI’s ability to rapidly infer customer sentiment through emotion recognition technology introduces both efficiency gains and ethical dilemmas. Sheng and Wang (2022) discuss how AI-driven sentiment analysis can enhance personalized service delivery, yet it also raises concerns regarding emotional manipulation and data privacy. If AI constantly monitors customer expressions, tone, and behavior, it must adhere to strict ethical guidelines to prevent exploitative practices.

At a broader level, Derave et al. (2022); Manjarrés et al. (2021) and Manjarrés et al. (2021) emphasize the risk of AI-enabled discrimination when rapid inference mechanisms are applied to immigration systems, job hiring processes, and consumer profiling. The speed at which AI processes and categorizes individuals may result in unfair outcomes, reinforcing systemic inequalities. This aligns with concerns Auld et al. (2022) raised regarding the governance of AI ethics, where rapid AI decision-making requires ongoing regulatory oversight to prevent harm.

Through the lens of belief as rapid discovery, ethical concerns emerge when AI systems generate and act upon information faster than it can be adequately verified or challenged. This helps explain issues related to misinformation, trust, security, and safety identified in this review. Consequently, rapid AI inference increases the importance of oversight mechanisms that ensure accuracy, accountability, and responsible decision-making.

These oversight concerns point to a deeper issue: autonomy itself can be reframed epistemically. Respect for autonomy is a core principle of AI ethics (Floridi and Cowls, 2019), yet AI systems in hospitality routinely act on inferred beliefs about guests, such as predicted preferences, willingness to pay, or likelihood of defection (Dursun-Cengizci and Caber, 2025), without the guest’s knowledge or ability to contest them. When these inferences shape which rooms, prices, or destinations a traveler ever sees, they bypass rational deliberation rather than engage it, a subtle form of manipulation (Susser et al., 2019; André et al., 2018). This is doxastic wronging in operational form: the system’s unjustified belief about a guest becomes a decision imposed on them. The “off-the-loop” condition described by Morosan and Dursun-Cengizci (2024) worsens the harm because neither guests nor employees can override the system, and its opacity prevents the belief from being examined or corrected ((Laitinen and Sahlgren, 2021)). Protecting autonomy therefore requires more than consent forms. It requires contestability, meaning ways for guests to see, question, and correct the inferences held about them, plus real human override at the service encounter (Tussyadiah, 2020). In an industry built on host and guest trust, this is a commercial need as much as an ethical one.

Academic integrity concerns can likewise be understood as epistemic failures, not just procedural violations. When scholars or students present AI-generated text as their own reasoning, they assert claims whose justification they do not hold, breaching the principle that one should not profess beliefs without adequate evidence (Clifford, 1877; Chignell, 2018). Generative AI worsens this because its outputs are fluent but not verified, producing plausible claims and fabricated citations (Dwivedi et al., 2023). For hospitality and tourism, this creates a dual risk: erosion of students’ critical thinking (Skavronskaya et al., 2023) and contamination of the field’s knowledge base if unverified AI-assisted claims enter the literature (Dogru et al., 2024; Nautiyal et al., 2023). Detection tools alone are insufficient given their error rates (Perkins, 2023), so scholars increasingly recommend disclosure requirements and redesigned assessment instead of policing alone (Cotton et al., 2024; Eke, 2023). Journals and programs in the field should require transparent disclosure of AI use, keep verification responsibility with human authors, and assess the reasoning process rather than the text alone.

### 4.3 Assessing AI risks in hospitality and tourism: a sectoral ranking

We conducted a sectoral risk analysis using risk criteria adapted from the GDPR (Table 5). The classification includes four tiers: unacceptable, high risk, limited risk, and minimal risks.

### 4.4 Integrating AI ethics and epistemology in hospitality and tourism: a life cycle perspective

Integrating AI into hospitality and tourism raises critical questions about how AI forms, justifies, and applies beliefs in decision-making processes. The AI life cycle provides a structured lens for analyzing these concerns. We comprehensively examined each stage, problem definition, data collection, model development, deployment, and feedback, both epistemological and ethical belief concerns, and proposed solutions to address them (Table 6).

## 5 Conclusion and implications

Integrating AI into hospitality and tourism presents a fundamental challenge: balancing operational efficiency with ethical responsibility. In this study, we analyzed the current notion of AI in hospitality and tourism through three epistemic lenses: belief as information, the tractable logic approach to belief, and belief as what one could rapidly discover. Our findings illustrate AI’s dual role as a rational decision-making agent and a potential source of ethical concern.

AI as information necessitates transparent and unbiased data management to prevent misinformation, privacy violations, and discriminatory practices. AI under tractable logic highlights the limitations of rigid decision-making frameworks, which, while efficient, may overlook ethical complexities in hospitality service delivery. AI as rapid inference presents critical risks regarding misinformation, surveillance, and biased decision-making, necessitating robust governance mechanisms to ensure accountability and fairness.

As Vidgen et al. (2020); Bickley and Torgler (2023) suggest, the ethical adoption of AI requires continuous assessment of its epistemic foundations, ensuring that belief formation processes align with ethical, legal, and societal expectations. AI’s role in hospitality and tourism will continue to evolve, but without ethical oversight, it risks compromising fairness, transparency, and consumer trust.

This study contributes to AI ethics research in three ways. First, it synthesizes the fragmented literature on AI ethics in hospitality and tourism into nine recurring ethical themes. Second, it advances a conceptual contribution by demonstrating that many ethical concerns originate from epistemological challenges associated with how AI systems generate, justify, and act upon beliefs about customers, employees, and service environments. Through this lens, issues such as bias, misinformation, privacy violations, and opacity can be understood as interconnected manifestations of flawed knowledge formation processes. Third, the study translates these insights into practical governance tools through a sector-specific risk framework and an AI lifecycle framework that support responsible AI implementation across hospitality and tourism contexts.

Additionally, this study advances ethical risk assessment frameworks by applying them specifically to AI applications in hospitality and tourism. It highlights industry-specific vulnerabilities, such as biases in AI-driven customer service, surveillance ethics in hotels, and the ethical implications of AI replacing human workers. It also bridges the gap between ethical AI theory and practice by proposing actionable solutions tailored to the tourism and hospitality industry’s unique structure, providing a roadmap for responsible AI deployment.

By integrating ethical theories with the practical realities of AI implementation, this research also offers novel solutions for overcoming the unique ethical dilemmas that hospitality and tourism face. These contributions lay a foundation for future studies, encouraging further exploration of the ethical dimensions of AI and providing practical strategies that can guide the deployment of AI in ethically responsible ways.

### 5.1 Limitations and future research

This study has several limitations. First, the search covered three databases; other databases may contain additional relevant studies. Second, only English-language, peer-reviewed articles were included, which may introduce language and publication bias. Third, the review focused on hospitality and tourism, excluding adjacent domains such as healthcare, so cross-domain comparisons remain for future work. Fourth, coding was performed by two researchers in a single round. Although disagreements were resolved through discussion, additional coders or a second coding round could further strengthen reliability, and the mapping of themes onto epistemological concepts inevitably reflects the authors’ interpretive choices. Future research should replicate and extend this synthesis as literature grows.

## Data Availability

The original contributions presented in the study are included in the article/Supplementary material, further inquiries can be directed to the corresponding author.
