# Supplementary material for: AI ethics in hospitality and tourism: theoretical perspectives, ethical beliefs, and actionable outcomes
Source: Front Artif Intell. 2026 Aug 14;9:1913343. doi: 10.3389/frai.2026.1913343 (PMC13522132; doi:10.3389/frai.2026.1913343)
Supplement: Supplementary file 1 [file Table_1.DOCX]

A.1: Search Query

| **Artificial Intelligence** | **“Artificial Intelligence” OR “AI” OR “Machine Learning” OR “ML” OR “Deep Learning” OR “Artificial Neural Networks” OR “Computer Vision”** |
| --- | --- |
| **Hospitality and Tourism** | “Hospitality and Tourism” OR “Hospitality” OR “tourism” OR “travel” OR “event management” OR “event” OR “guest services” OR “food and beverage” OR “leisure” OR “accommodation” OR “tourist” OR “hotel” OR “airbnb” OR “lodging” OR “cruise” OR “theme park” OR “restaurant” or “casino” or “gambling” |
| **Ethics** | “Ethics” OR “Fairness” OR “Integrity” OR “Virtues” OR “Value-System” OR “Ethical Values” OR “Rightness” OR “Moral” OR “Morality” |
| **Guidelines** | “Guidelines” OR “Recommendations” OR “Instructions” OR “requirements” OR “principles” OR “regulations” OR “suggestions” OR “advice” OR “Rules” OR “standard” OR “criteria” |
| **Barriers** | “resistance” OR  “barriers” OR  “limitations” OR “challenges” |

A.2: Privacy and Data Management

| **Authors** | **Year** | **Journal** | **Methodology** |
| --- | --- | --- | --- |
| Morosan, C. and Dursun-Cengizci, A. | 2024 | International Journal of Contemporary Hospitality Management | Quantitative |
| Hsu, C.H.C. et al. | 2024 | Annals of Tourism Research | Literature Review |
| Bulchand-Gidumal, J. et al. | 2023 | Current Issues in Tourism | Qualitative |
| Zou, T. et al.. | 2022 | Geographical Research | Quantitative |
| Hadjistavropoulos, T. | 2023 | Canadian Psychology | Literature Review |
| Derave, C. et al. | 2022 | European Journal of Risk Regulation | Literature Review |
| Oravec, J.A. | 2021 | Change Management | Qualitative |
| Vidgen, R et al. | 2020 | European Journal of Operational Research | Qualitative |

A.3: Bias and Discrimination

| **Authors** | **Year** | **Journal** | **Methodology** |
| --- | --- | --- | --- |
| Lin, BY et al. | 2023 | Journal of Hospitality and Tourism Research | Qualitative |
| Auld, G. et al. | 2022 | Journal of European Public Policy | Literature review |
| Derave, C. et al.. | 2022 | European Journal of Risk Regulation | Literature review |
| Zebrowski, R. | 2021 | Pro-Fil | Literature review |
| Mandez-Suarez, M et al. | 2023 | Journal of Competitiveness | Quantitative |
| Manjarras, A et al. | 2021 | IEEE Technology and Society Magazine | Literature review |
| W Orr, and JL Davis | 2020 | Information, Communication & Society | Qualitative |
| A Mamychev et al. | 2021 | Wisdom | Literature review |
| Azzutti et al. | 2021 | University of Pennsylvania Journal of International Law | Literature review |
| Dogru et al. | 2023 | Tourism Economics | Quantitative |

A.4: Misinformation and Trust

| **Author** | **Year** | **Journal** | **Methodology** |
| --- | --- | --- | --- |
| Nautiyal, R et al. | 2023 | Annals of Tourism Research | Literature review |
| Hsu, C.H.C et al. | 2024 | Annals of Tourism Research | Literature review |
| Dogru, T. et al. | 2023 | Tourism Economics | Quantitative |
| Zebrowski, R. | 2021 | Pro-Fil | Literature review |
| Roestamy, M et al. | 2023 | Journal of Engineering Science and Technology | Quantitative |
| Skavronskaya et al. | 2023 | Journal of Teaching in Travel and Tourism | Qualitative |
| M Crippen | 2023 | Synthese | Qualitative |
| W Orr, JL Davis | 2020 | Information, Communication & Society | Qualitative |
| Mateo, J.F. | 2022 | International Visual Culture Review | Qualitative |
| Stohy, A. et al. | 2021 | PLoS ONE | Quantitative |
| Sop, SA and Kuraser, D | 2024 | Journal of Hospitality and Tourism Technology | Quantitative |
| Bhaskar, P and Sharma, KD | 2022 | Pacific Business Review International | Qualitative |

A.5: Impact on Society

| **Author** | **Year** | **Journal** | **Methodology** |
| --- | --- | --- | --- |
| Arvan, M | 2023 | AI & Society | Literature review |
| Lin, BY et al. | 2023 | Journal of Hospitality and Tourism Research | Qualitative |
| Sheng, A. and Wang, F. | 2022 | Neohelicon | Literature review |
| Kim, J.H. et al. | 2023 | Journal of Travel and Tourism Marketing | Quantitative |
| I Shklovski, C Némethy | 2023 | Information, Communication & Society | Qualitative |
| Azzutti et al. | 2021 | University of Pennsylvania Journal of International Law | Quantitative |
| A Mamychev et al. | 2021 | Wisdom | Literature review |
| Jaillant, L and Caputo, A | 2022 | AI & Society | Qualitative |

A.6: Transparency and Fairness

| **Authors** | **Year** | **Journal** | **Methodology** |
| --- | --- | --- | --- |
| Lin, BY et al. | 2023 | Journal of Hospitality and Tourism Research | Qualitative |
| Chon, K.K.S. and Hao, F. | 2024 | Tourism Review | Literature review |
| Hadjistavropoulos, T. | 2023 | Canadian Psychology | Literature review |
| Oravec, J.A. | 2021 | Change Management | Qualitative |
| Stohy, A. et al. | 2021 | PLoS ONE | Quantitative |
| Mandez-Suarez, M et al. | 2023 | Journal of Competitiveness | Quantitative |
| Bickley, SJ and Torgler, B | 2023 | AI & Society | Literature review |
| Manjarras, A et al. | 2021 | IEEE Technology and Society Magazine | Literature review |
| Bhaskar, P & Sharma, KD | 2022 | Pacific Business Review International | Qualitative |
| Vidgen, R et al. | 2020 | European Journal of Operational Research | Literature review |

A.7: Autonomy and Responsibility

| **Author** | **Year** | **Journal** | **Methodology** |
| --- | --- | --- | --- |
| Morosan, C. and Dursun-Cengizci, A. | 2024 | International Journal of Contemporary Hospitality Management | Quantitative |
| Mateo, J.F. | 2022 | International Visual Culture Review | Qualitative |
| Bhaskar, P and Sharma, KD | 2022 | Pacific Business Review International | Qualitative |

A.8: Ethics in AI Development

| **Author** | **Year** | **Journal** | **Methodology** |
| --- | --- | --- | --- |
| Bulchand-Gidumal, J. et al. | 2023 | Current Issues in Tourism | Qualitative |
| Auld, G. et al. | 2022 | Journal of European Public Policy | Qualitative |
| Chen, C. et al.. | 2024 | Heliyon | Quantitative |
| Bickley et al. | 2023 | AI & Society | Literature review |
| Manjarras, A et al. | 2021 | IEEE Technology and Society Magazine | Literature review |
| Jaillant, L and Caputo, A | 2022 | AI & Society | Qualitative |
| Clayton, J and Sibbald, S | 2020 | International Review of Information Ethics | Literature review |
| A Mamychev, E et al. | 2021 | Wisdom | Literature review |

A.9: Security and Safety

| **Authors** | **Year** | **Journal** | **Methodology** |
| --- | --- | --- | --- |
| Lin, BY et al. | 2023 | Journal of Hospitality & Tourism Research | Qualitative |
| Kim, J.H. et al. | 2023 | Journal of Travel and Tourism Marketing | Quantitative |
| Kim, M.J. et al. | 2022 | Journal of Travel Research | Quantitative |
| Mateo, J.F | 2022 | International Visual Culture Review | Qualitative |
| Oravec, J.A | 2011 | Change Management | Qualitative |
| Roestamy, M et al. | 2023 | Journal of Engineering Science and Technology | Quantitative |
| Mandez-Suarez et al. | 2023 | Journal of Competitiveness | Quantitative |

A.10. Academic Integrity

| **Author** | **Year** | **Journal** | **Methodology** |
| --- | --- | --- | --- |
| Nautiyal, R et al. | 2023 | Annals of Tourism Research | Literature review |
| Arvan, M | 2023 | AI & Society | Literature review |
| Dogru, T. et al. | 2023 | Tourism Economics | Literature review |

A.11: Mapping Findings Based on the Epistemology of AI in Hospitality and Tourism

| **Epistemology of AI Framework** | **Relevant Findings from Hospitality and Tourism** | **Examples in Hospitality and Tourism** |
| --- | --- | --- |
| **Sources of AI Belief Formation** | **Bias and Discrimination**: AI systems rely on biased datasets, leading to unjustified beliefs about guest preferences or behavior. | Hotel recommendation systems suggesting premium packages exclusively to guests with certain demographics. |
|  | **Privacy and Data Management**: Inadequate sources for belief formation compromise guest privacy and lead to unjustified AI recommendations. | AI predicting guest preferences by mining private data without consent. |
| **Justification of AI Beliefs** | **Transparency and Fairness**: Lack of explainability in AI beliefs can result in unjustified actions impacting guest satisfaction and trust. | AI assigning room upgrades without explaining the criteria, leading to perceptions of unfairness. |
|  | **Misinformation and Trust**: AI beliefs must be justified to prevent the spread of incorrect or harmful information. | AI promoting "safe" destinations that are actually unsafe due to outdated crime data. |
| **Knowledge vs. Belief in AI Systems** | **Impact on Society**: Distinguishing between what AI knows and what it assumes is critical in travel decision-making systems. | AI assuming a guest prefers luxury services without verifying actual preferences. |
|  | **Autonomy and Responsibility**: AI systems should differentiate between actionable knowledge and speculative beliefs. | AI suggesting activities based on incomplete data, undermining guest autonomy in decision-making. |
| **Epistemic Reliability of AI** | **Security and Safety**: The reliability of AI beliefs is vital to ensuring guest safety in tourism and hospitality contexts. | AI surveillance in hotels must accurately identify security risks without profiling. |
|  | **Ethics in AI Development**: Ensuring the reliability of AI beliefs through rigorous testing and ethical oversight. | AI hiring systems must reliably assess candidates without embedding bias. |
| **Epistemic Opacity** | **Transparency and Fairness**: Guests may mistrust AI systems that operate as "black boxes" without revealing how beliefs are formed. | Recommender systems that suggest activities without explaining their algorithmic logic. |
|  | **Misinformation and Trust**: Lack of transparency about belief formation processes fosters mistrust in AI travel tools. | AI-generated itineraries with vague justifications for included destinations. |
| **Epistemic Injustice** | **Bias and Discrimination**: Marginalized groups may face epistemic injustice when their preferences are excluded from AI training data. | AI failing to accommodate dietary preferences or accessibility needs for certain groups. |
|  | **Privacy and Data Management**: Using guest data without consent perpetuates epistemic injustice, as it assumes unwarranted access to personal information. | AI systems predicting preferences without obtaining explicit guest consent for data use. |
| **Pragmatic Epistemology** | **Impact on Society**: Practical consequences of AI beliefs, such as job replacement and guest dissatisfaction, must be considered. | AI replacing human concierge roles without achieving equivalent levels of guest satisfaction. |
|  | **Moral Responsibility for AI Beliefs**: AI systems must be designed to prioritize practical outcomes, such as enhancing guest experiences ethically. | AI offering personalized recommendations that balance business goals and guest well-being. |
| **Epistemic Risk and Uncertainty** | **Misinformation and Trust**: AI systems in hospitality must account for the uncertainty in their beliefs and communicate this clearly to users. | AI suggesting weather-dependent activities without accounting for sudden changes in conditions. |
|  | **Security and Safety**: The potential risks of uncertain beliefs in AI systems, such as flawed safety recommendations, must be mitigated. | AI recommending underdeveloped destinations as "safe" based on incomplete data. |
